# High-throughput screening identifies modulators of sarcospan that stabilize muscle cells and exhibit activity in the mouse model of Duchenne muscular dystrophy

**DOI:** 10.1186/s13395-020-00244-3

**Published:** 2020-09-18

**Authors:** Cynthia Shu, Liubov Parfenova, Ekaterina Mokhonova, Judd R. Collado, Robert Damoiseaux, Jesus Campagna, Varghese John, Rachelle H. Crosbie

**Affiliations:** 1 Molecular Biology Institute, University of California Los Angeles, Los Angeles, CA USA; 2 Department of Integrative Biology and Physiology, University of California Los Angeles, 610 Charles E. Young Drive East, Terasaki Life Sciences Building, Los Angeles, CA 90095 USA; 3 Center for Duchenne Muscular Dystrophy, University of California Los Angeles, Los Angeles, CA USA; 4 Department of Molecular and Medicinal Pharmacology, University of California Los Angeles, Los Angeles, CA USA; 5 California NanoSystems Institute, University of California Los Angeles, Los Angeles, CA USA; 6 Department of Neurology, David Geffen School of Medicine, University of California Los Angeles, 610 Charles E. Young Drive East, Terasaki Life Sciences Building, Los Angeles, CA 90095 USA; 7 Drug Discovery Lab, University of California Los Angeles, Los Angeles, CA USA

**Keywords:** Drug discovery, Duchenne muscular dystrophy, Dystrophin, High-throughput screen, Sarcolemma, Sarcospan, Small molecules

## Abstract

**Background:**

Duchenne muscular dystrophy (DMD) is a degenerative muscle disease caused by mutations in the dystrophin gene. Loss of dystrophin prevents the formation of a critical connection between the muscle cell membrane and the extracellular matrix. Overexpression of sarcospan (SSPN) in the mouse model of DMD restores the membrane connection and reduces disease severity, making SSPN a promising therapeutic target for pharmacological upregulation.

**Methods:**

Using a previously described cell-based promoter reporter assay of SSPN gene expression (hSSPN-EGFP), we conducted high-throughput screening on libraries of over 200,000 curated small molecules to identify SSPN modulators. The hits were validated in both hSSPN-EGFP and hSSPN-luciferase reporter cells. Hit selection was conducted on dystrophin-deficient mouse and human myotubes with assessments of (1) SSPN gene expression using quantitative PCR and (2) SSPN protein expression using immunoblotting and an ELISA. A membrane stability assay using osmotic shock was used to validate the functional effects of treatment followed by cell surface biotinylation to label cell surface proteins. Dystrophin-deficient *mdx* mice were treated with compound, and muscle was subjected to quantitative PCR to assess SSPN gene expression.

**Results:**

We identified and validated lead compounds that increased SSPN gene and protein expression in dystrophin-deficient mouse and human muscle cells. The lead compound OT-9 increased cell membrane localization of compensatory laminin-binding adhesion complexes and improved membrane stability in DMD myotubes. We demonstrated that the membrane stabilizing benefit is dependent on SSPN. Intramuscular injection of OT-9 in the mouse model of DMD increased SSPN gene expression.

**Conclusions:**

This study identifies a pharmacological approach to treat DMD and sets the path for the development of SSPN-based therapies.

## Background

Duchenne muscular dystrophy (DMD) is a degenerative muscle disease that affects 1 in every 5700 males [1]. DMD is caused by mutations in the gene that encodes the dystrophin protein. Currently, the only Food and Drug Administration approved treatments for DMD are corticosteroids and exon skipping therapies. In clinical trials, corticosteroids extended the age of loss of ambulation by several years, and exon skipping therapies slowed disease progression, but neither is considered a cure [2, 3]. Although significant progress has been made in the development of DMD therapies, there remains a need for treatments that can improve the quality of life and life expectancy of individuals affected by DMD. Small molecules are the most common modality used to treat diseases due to ease of delivery and reduced risk for immune responses relative to viral or cell-based modalities. In this study, we report on the identification and characterization of small molecules that are predicted to benefit all DMD patients regardless of mutation. Lastly, we demonstrate the in vivo efficacy of a lead hit compound using the dystrophin-deficient *mdx* murine model for DMD.

In healthy muscle, dystrophin localizes to the intracellular surface of myofibers where it connects the actin cytoskeleton, cell membrane (sarcolemma), and extracellular matrix (ECM) to stabilize the muscle cell during contractions [4, 5]. Dystrophin associates with sarcospan (SSPN), the sarcoglycans, the dystroglycans, and numerous other proteins to form the dystrophin-glycoprotein complex (DGC) [6–9]. Within the DGC, dystrophin interacts directly with F-actin and β-dystroglycan, which associates with the laminin-binding protein, α-dystroglycan (α-DG) [10]. The DGC is one of three major adhesion complexes in muscle that serve to connect the myofiber to the ECM, and its presence is critical for proper muscle maintenance. In individuals with DMD, loss of dystrophin and the DGC leads to contraction-induced damage and muscle degeneration [11]. By the second to third decade of life, individuals with DMD experience severe muscle wasting that also affects the heart and respiratory system [1].

Along with the DGC, the utrophin-glycoprotein complex (UGC) and the α7β1D-integrin complex serve as adhesion complexes between the cell membrane and the ECM [12, 13]. The UGC localizes to neuromuscular junctions and has a similar composition to the DGC, with utrophin taking the place of dystrophin [14–16]. Extrasynaptic redistribution of the UGC is well documented in dystrophin-deficient muscle, suggesting the existence of a compensatory mechanism involving upregulation of endogenous adhesion complexes [17]. Decades of research have since established that transgenic upregulation of the UGC and α7β1D-integrin complex prevents muscle pathology in the *mdx* mouse model of DMD by compensating for the loss of the DGC [18, 19].

SSPN is a 25 kDa transmembrane protein that is expressed in striated muscle and associates with all three adhesion complexes [20–22]. Mutagenesis studies demonstrated that SSPN protein contains multiple oligomer interaction sites and that it forms a tight association with the sarcoglycans, suggesting that SSPN acts as a protein scaffold [21]. Transgenic overexpression of SSPN in *mdx* mice increased the membrane localization of the UGC and the α7β1D-integrin complex, leading to improved membrane stability, reduced muscle pathology, and improved muscle function [22–24]. In addition to the improvements in skeletal muscle, SSPN upregulation improved cardiac and respiratory function, the major cause of mortality among DMD patients [24, 25]. Importantly, 30-fold increased levels of SSPN did not induce detrimental side effects, demonstrating that SSPN therapies are both safe and effective in preclinical studies [24]. In the current study, we identified and characterized small molecule modalities to increase SSPN. Our results demonstrate that pharmacological targeting of SSPN produces therapeutic levels of SSPN protein in dystrophin-deficient myotubes.

## Methods

### Reporter cell lines

The human sarcospan EGFP reporter C2C12 cell line (hSSPN-EGFP) was used as described by Shu and colleagues [26]. Using an identical approach, a human sarcospan luciferase (hSSPN-luc) C2C12 cell line was created and used in secondary screening.

### Small molecules

Screening libraries were provided by the Molecular Screen Shared Resource at the University of California Los Angeles [27]. Commercially available compounds used in follow-up studies were purchased from Asinex and Life Chemicals Inc.

### High-throughput screening

hSSPN-EGFP myoblasts were seeded at 500 cells per well in 50 μl of growth medium in 384-well black, clear bottom microplates (Greiner) using a Multidrop 384 (Thermo Fisher Scientific) and incubated for 3 days to allow cells to reach confluency. Upon reaching confluency, the growth medium was replaced with 50 μl of differentiation medium consisting of DMEM with 2% horse serum (Sigma-Aldrich) using an EL406 combination washer dispenser (Biotek). At day 2 of differentiation, the medium on the cells was aspirated, left with a residual volume of 10 μl, and replaced with 30 μl of fresh differentiation medium. Following media replacement, 0.5 μl of small molecule in DMSO or DMSO alone (for vehicle and positive control wells) was added to each well using a Biomek Fx (Beckman). To ensure proper mixing of the DMSO, 50 μl of additional differentiation medium was added to all wells except the positive control treated wells, which instead received 50 μl of medium-containing insulin transferrin selenium (ITS) (Gibco) to reach a final concentration of 1% ITS. The final concentration of compound in each treated well was 5.5 μM in 0.55% DMSO and 0.55% DMSO only for vehicle and positive control treated wells. After 48 h of incubation, the medium was replaced with Fluorobrite DMEM (Gibco), and each plate was imaged using ImageXpress Micro Confocal High Content Imaging System (Molecular Devices). The fluorescence intensity of imaged cells was determined using a custom module analysis in MetaXpress Analysis software (Molecular Devices). Analysis setting were as follows: top hat (size: 12, filter shape: circle), adaptive threshold (source: top hat, minimum width: 10, maximum width: 800, intensity above local background: 500), filter mask (filter type: minimum area filter, minimum value: 500).

### Luciferase assay

hSSPN-luciferase myoblasts were cultured as described above. After 48 h of treatment, plates were allowed to equilibrate to RT. The cell culture medium in each well was aspirated using an EL406 combination washer dispenser. Bright-Glo luciferase assay system reagent (Promega) and differentiation medium were added to cells at a 1:2 dilution using a Multidrop 384. After a 3-min incubation at RT, luminescence signal was quantified using an Envision plate reader (PerkinElmer). The relative luminescence units were analyzed to determine fold change of treated over vehicle-treated cells.

### Cell culture

C2C12 cells (American Type Culture Collection) were grown at 37 °C with 5% CO_2_ in growth medium containing DMEM (Gibco) with 20% FBS (Sigma-Aldrich). Upon reaching 90–100% confluency, myoblasts were induced to differentiate by replacing the medium with differentiation medium consisting of DMEM with 2% horse serum (Sigma-Aldrich). Conditionally immortalized H2K WT and *mdx* myoblasts with a nonsense mutation in exon 23 of dystrophin were a gift from Terrance Partridge, Ph.D. (Children’s National Medical Center, Washington, D.C.) [28]. Myoblasts were allowed to proliferate on 0.01% gelatin (Sigma-Aldrich)-coated plates at 33 °C with 5% CO_2_ with growth medium containing DMEM, 20% HI-FBS (Invitrogen), 2% l-glutamine (Sigma-Aldrich), 2% chicken embryo extract (Accurate Chemical), 1% penicillin-streptomycin (Sigma-Aldrich), and 20 U/ml of fresh interferon gamma (Gibco). For differentiation, H2K myoblasts were seeded on plates coated with 0.1 mg/ml matrigel (Corning) diluted in DMEM and grown in proliferation conditions. Upon reaching 90–100% confluency, cells were grown at 37 °C with 5% CO_2_ in differentiation medium containing DMEM with 5% horse serum (Sigma-Aldrich), 2% l-glutamine, and 1% penicillin-streptomycin using established protocols. The immortalized human DMD myoblast cell line was grown in Skeletal Muscle Basal Medium (Promocell) containing 20% FBS (Fisher Scientific), Skeletal Muscle Growth Supplement Mix (Promocell), and 1% penicillin-streptomycin at 37 °C with 5% CO_2_ [29]. Upon, reaching 100% confluency, the medium was replaced with differentiation medium containing Skeletal Muscle Basal Medium, Skeletal Muscle Differentiation Supplement Mix (Promocell), and 1% penicillin-streptomycin.

### Gene expression analysis

RNA from myotubes treated for 48 h was extracted from cells using Trizol-based (Thermo Fisher Scientific) phase separation, as previously described [30]. RNA concentrations were determined using a NanoDrop 1000 (Thermo Fisher Scientific), and 750 ng of RNA in a 20 μl reaction was reverse transcribed using iScript cDNA synthesis (Bio-Rad) with the following cycling conditions: 25 °C for 5 min, 42 °C for 30 min, 85 °C for 5 min. For mouse qPCR, SsoFast EvaGreen Supermix (Bio-Rad), 400 nM of each optimized forward and reverse primer (SSPN F: 5’ TGCTAGTCAGAGATACTCCGTTC 3’, SSPN R: 5’ GTCCTCTCGTCAACTTGGTATG 3’, BACT F: 5’ GAGCACCCTGTGCTGCTCACCG 3’, BACT R: 5’ CAATGCCTGTGGTACGACCA 3’), and cDNA corresponding to 37.5 ng RNA were used to amplify cDNA measured by QuantStudio 5 Real-Time PCR System (Thermo Fisher Scientific) with the following reaction conditions: 55 °C for 2 min, 95 °C for 2 min, 40 cycles of 95 °C for 10 s and 62 °C for 30 s, and dissociation stage. For qPCR of human samples, TaqMan assays were used to quantify SSPN (assay ID Hs01025520m_1) and ACTB (assay ID Hs01060665_g1) with the following reaction conditions: 50 °C for 2 min, 95 °C for 10 min, 40 cycles of 95 °C for 15 s and 62 °C for 1 min. Each sample was run in triplicate. Data was analyzed using the ddCT method and normalized to reference gene ACTB with vehicle-treated samples serving as the calibrator (relative expression of vehicle control = 1).

### Immunoblotting

Myotubes treated for 48 h were lysed using RIPA buffer (Thermo Fisher Scientific) containing Halt Protease and Phosphatase Inhibitor Cocktail (Thermo Fisher Scientific). Cell lysates in RIPA buffer were rocked for 1 h at 4 °C and centrifuged at 1000 RPM for 30 min at 4 °C. The supernatant was collected, quantified for protein concentration using the DC protein assay (Bio-Rad), and normalized to 2 mg/ml in water and Laemmli sample buffer with a final concentration of 10% glycerol (Sigma-Aldrich), 5% beta-mercaptoethanol (Sigma-Aldrich), 3% sodium dodecyl sulfate (Sigma-Aldrich), and 0.05% bromophenol blue (Sigma-Aldrich). For SDS-PAGE, samples were heated to 95 °C for 2 min before loading 40 μg onto 4–12% Tris-glycine or Bis-Tris polyacrylamide gels (Novex), electrophoresed for 2 h at 100 volts at RT, and transferred to a nitrocellulose membrane for 2 h at 100 volts at 4 °C. Ponceau S staining was performed to visualize protein loading and verify protein transfer. Membranes were blocked with 5% nonfat dried milk in Tris-buffered saline pH 7.4 with 0.1% tween-20 (Sigma-Aldrich) (TBST) for 1 h at RT and incubated on a rocker overnight at 4 °C with the following primary antibodies diluted in blocking buffer containing 5% nonfat dry milk (Carnation) unless otherwise noted: SSPN (sc-393187, Santa Cruz Biotechnology, 1:200), glycosylated alpha-dystroglycan (IIH6 C4, Developmental Studies Hybridoma Bank, 1:100 in 1% milk, core alpha-dystroglycan (Beadle Lab, 1:1000 in 1% milk), UTRN (MANCHO3, Developmental Studies Hybridoma Bank, 1:100), and GAPDH (Mab374, Millipore, 1:10,000). Following three 10-min TBST washes, the membranes were incubated in goat antimouse IgG HRP (ab6789, Abcam, 1:5000 for all, 1:10,000 for GAPDH in 5% milk) or goat antirabbit IgG HRP (ab6721, Abcam, 1:10,000 in 1% milk) for 1 h at RT. The membranes were then washed three times for 10 min each with TBST, incubated in SuperSignal West Pico Chemiluminescent Substrate (Thermo Fisher Scientific) for 5 min at RT on an orbital shaker, and exposed to autoradiography films (Agfa). Autoradiography films were developed using a SRX-101A tabletop processor (Konica Minolta), scanned to a digital file, and analyzed by densitometry of bands using ImageJ version 1.51 s [31]. Target protein bands were normalized to loading control GAPDH with vehicle-treated cells serving as the calibrator sample (relative protein levels of vehicle control = 1).

### ELISA to quantify SSPN protein levels

DMD myotubes were lysed in modified RIPA buffer containing 1% Triton X-100, 0.05% DOC, 0.05% SDS, and Halt protease and phosphatase inhibitors and subjected to 3 rounds of sonication. The lysate was centrifuged at 12,000×*g* for 10 min, and the supernatant was transferred to new tubes. Protein concentration was determined using DC assay. A 96-well Nunc MaxiSorp plate (Invitrogen) was coated with 100 μl of 1 μg/ml SSPN antibody (sc-393187, Santa Cruz Biotechnology) per well overnight at 4 °C, washed three times with 1X TBS containing 0.05% Tween-20, blocked for 1 h at RT in 1% bovine serum albumin (BSA), and washed again. Recombinant human SSPN standards (100 μl; Novus Biologicals) and lysates were added to plate, incubated overnight at 4 °C, and washed. SSPN antibody (Life Span Biosciences, LS-C747357, 100 μl of 400 ng/ml) was added to each well and incubated for 2 h at RT and washed. Goat antirabbit IgG HRP (Abcam, 1:10,000, 100 μl) was added to each well, incubated for 2 h at RT, and washed. After the wash, 100 μl of tetramethylbenzidine (Fisher Scientific) was added to each well and incubated for 30–40 min RT. After signal development, 100 μl of stop solution was added to each well, and the absorbance at 450 nm was measured for each well using a plate reader the SpectraMax M2 microplate reader (Molecular Devices). The concentration of SSPN protein for each sample was calculated using the standard curve and normalized to protein concentration and vehicle-treated controls.

### Analysis of cell surface proteins

After 48 h of treatment, myotubes were washed with ice cold PBS containing 0.1 g/L of both CaCl_2_ (0.9 mM) and MgCl_2_ (1.05 mM) (Corning) three times and incubated in 0.5 mg/ml of EZ-Link Sulfo-NHS-SS-Biotin (Thermo Fisher Scientific) at 4 °C with gentle rotation for 30 min to label cell surface proteins. All steps were performed at 4 °C unless otherwise mentioned. The cells were washed three times with ice cold 100 mM glycine in PBS for 5 min with gentle rotation to remove nonreacted biotin. Following a PBS wash, the cells were lysed in solubilization buffer composed of 50 mM Tris-HCl pH 7.8, 500 mM NaCl, 1% digitonin (Biosynth), and Halt protease and phosphatase inhibitors. The samples were rotated 4 °C for 10 min and centrifuged at 4 °C at 14,000 rpm for 20 min to pellet debris. The DC assay (Bio-Rad) was used to determine the protein concentration of the supernatant (total lysate). Pierce High Capacity Neutravidin Agarose (Thermo Fisher Scientific) beads were washed with solubilization buffer before being combined with equal concentrations of total lysate and incubated at 4 °C overnight with rotation. The beads were centrifuged at 4 °C at 2500 rpm for 5 min and washed with solubilization buffer containing 0.1% digitonin. This was repeated for a total of 4 washes. The biotinylated cell surface proteins were cleaved from biotin-avidin using 2 x Laemmli sample buffer (LSB) with 50 mM DTT, rotated at RT for 60 min, and heated at 95 °C for 5 min. The samples were centrifuged at 2500 rpm at 4 °C for 5 min, and the supernatant (membrane fraction) was collected for immunoblot analysis.

### Membrane stability assay

The membrane stability assay was modified from previously described methods [32]. The solutions for osmotic shock were prepared from a base solution containing 5 mM HEPES, 5 mM KCl, 1 mM MgCl_2_, 5 mM NaCl, 1.2 mM CaCl_2_, and 1 mM glucose. Sucrose was added to the base solutions to reach osmolarities of 50, 80, 100, 280, and 300 mosmol. The actual osmolarity was determined using a VAPRO vapor pressure osmometer (Wescor Inc.). Myotubes were treated for 48 h and at day 4 of differentiation were subjected to 20 min of osmotic shock at 37 °C using 28.5 to 223.5 milliosmole (mosmol) solutions. The supernatant was collected and centrifuged to separate cell debris. Adherent cells were trypsinized and pelleted before lysis with water and 3 freeze-thaw cycles. The Creatine Kinase Assay (Sekisui Diagnostics) was used to measure creatine kinase (CK) levels in both the supernatant and lysate fractions. In a 96-well plate, 4 μl of each sample and 140 μl of reagent was loaded per well in triplicate. The U/L of CK was calculated as follows: (mOD/min) (total volume in mL) (dilution factor)/(6.22 M^−1^ cm^−1^) (light path in cm) (sample volume in mL). The percent CK release was calculated as follows: CK_extracellular_/(CK_extracellular_ + CK_intracellular_) * 100.

### siRNA-mediated knockdown

Lipofectamine RNAiMAX Transfection Reagent (Life Technologies) was used to transfect H2K *mdx* myotubes with 24 or 48 nM of Silencer Select SSPN siRNA (siRNA ID s68932, Life Technologies) or MISSION siRNA Fluorescent Universal Negative Control #1, Cyanine 3 (Sigma Aldrich) diluted in Opti-MEM Reduced Serum Medium (Thermo Fisher Scientific). The transfection reagent and diluted siRNA were added to 1 ml of growth medium per well in a 24-well cell culture plate.

### Myotube fusion index

Myoblasts in a 96-well plate were treated for 72 h beginning at day 2 of differentiation were fixed with 4% paraformaldehyde for 20 min, permeabilized with 0.2% Triton X-100 (Sigma) for 10 min, and blocked with 1% BSA for 30 min. Myosin heavy chain (MHC) was detected using 10 μg/ml MF-20 (Developmental Hybridoma Studies Bank) in 1% BSA overnight and 10 μg/ml goat antimouse Alexa Fluor Plus 594 (Thermo Fisher Scientific) in 1% BSA for 1 h. PBS washes were performed between each step above. Nuclei were stained with 5 μg/ml Hoechst (Thermo Fisher Scientific) for 20 min before imaging. Each treatment was performed in three wells, and three fields per well were captured. ImageJ was used to count the number of total nuclei and nuclei within a MHC positive cell. Fusion index was calculated as nuclei in a MHC positive cell/total nuclei.

### Half-life analysis

Half-life analysis was performed by Eurofins Panlabs Inc. using 1 μM of compound with a final DMSO concentration of 0.5%. PBS or plasma from CD-1 mice was prewarmed to 37 °C for 5 min before addition of the test compounds and continued incubation at 37 °C. At 0, 30, 60, 120, 240, and 1440 min, an aliquot of solution containing compounds was mixed with acetonitrile/methanol, mixed, and centrifuged. The supernatants were used for HPLC-MS/MS analysis.

### In vivo treatment

For the preliminary safety assessment of OT-9, we performed IP injections in 6-month-old C57/Bl6 males. The mice were injected with 100 μl of vehicle (5% DMSO (Sigma), 95% PBS), 100 μl of 30 mg/kg of OT-9 in vehicle, or 100 μl of 50 mg/kg of OT-9 in vehicle (*n* = 1 mouse per treatment). The mice were observed for 72 h, then sacrificed for visual evaluation of injection site and internal organs (data not shown). For assessment of activity, 20-week-old male *mdx* littermates were injected in both tibialis anterior muscles with vehicle (5% DMSO, 95% PBS) or 86 μg of OT-9. Two mice were injected in both TAs with vehicle, and three mice were injected in both TAs with 20 μl of OT-9 (9.4 mM solution containing 86 μg of OT-9). After 4 h, the muscles were harvested and processed for gene expression analysis.

### Data analysis

Robust strictly standardized mean difference (SSMD*) was used to assess plate quality and for hit selection. SSMD* = *X*_*P*_ − *X*_*N*_/1.4826$\sqrt{\ {s}_P^2+{s}_N^2\ }$, where *X*_*P*_, *X*_*N*_, *S*_*P*_, and *S*_*N*_ are the medians and median absolute deviations of the positive and negative controls, respectively [33]. For plate quality, SSMD* ≥ 1 indicates a good quality moderate positive control. For initial hit selection, a 1.4-fold increase over vehicle and SSMD* > 0.25 was considered a hit. Statistical analysis was performed using Prism version 7.0 (GraphPad Software) for Mac OS X using the two-tailed, non-parametric Kolmogorov-Smirnov test. Data are reported as mean + SEM. A *p* value of < 0.05 was considered statistically significant, *p < 0.05, **p < 0.01, ***p < 0.001, and ****p < 0.0001.

## Results

We previously created and validated a muscle cell–based high-throughput assay to identify small molecule enhancers of human SSPN gene expression [26]. Using the assay, we screened clinical compounds and demonstrated that the assay is capable of identifying small molecules that increase SSPN gene and protein expression in both wild-type and dystrophin deficient myotubes [26]. In this current study, we screened large chemical libraries with the goal of identifying compounds that can be developed into new chemical entity enhancers of SSPN. The curated libraries were developed to maximize drug-likeness based on Lipinski’s rule of 5, which defines parameters that can be used to predict optimal oral bioavailability in humans [27].

### High-throughput screening of 200,000 small molecules

**Fig. 1:**
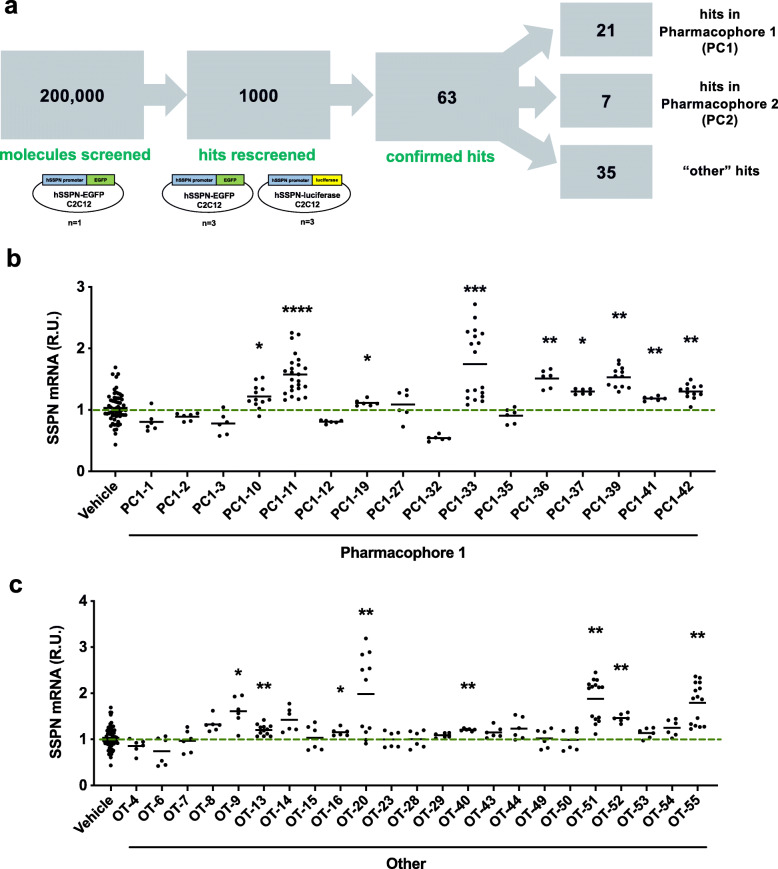
Pipeline for high-throughput screening for sarcospan modulators. **a** Large chemical libraries were screened using the hSSPN-EGFP C2C12 reporter cell line (*n* = 1). The top 1000 hits were rescreened in both hSSPN-EGFP and hSSPN-luciferase C2C12 reporter cell lines (*n* = 3 each). Sixty-three of 1000 compounds increased reporter expression in both cell lines and were therefore considered confirmed hits. The confirmed hits were sorted into 3 groups based on common structural features: pharmacophore 1 (PC1), pharmacophore 2 (PC2), and other, which had no unifying structural theme. Dystrophin-deficient H2K *mdx* cells were treated with 5.5 μM of **b** pharmacophore 1 (PC1) or **c** other compounds for 48 h. All cells were treated at day 2 of differentiation and harvested 48 h posttreatment. Gene expression was normalized to housekeeping gene β-actin and vehicle-treated cells (0.5% DMSO). Data represents individual replicates and mean value. *n* = 3–8. SSPN, sarcospan; R.U., relative units. *p < 0.05, **p < 0.01, ***p < 0.001, ****p < 0.0001

High-throughput screening of over 200,000 small molecules from curated libraries was conducted using a cell-based assay for human SSPN gene expression. The reporter cells used in the assay were C2C12 murine myoblasts stably transfected with a construct containing the human SSPN promoter region followed by the coding sequence for enhanced green fluorescent protein (hSSPN-EGFP). Using the hSSPN-EGFP assay, we screened compounds at a concentration of 5.5 μM (*n* = 1) (Fig. 1a). Plate quality was calculated using robust strictly standardized mean difference (SSMD*) (Additional file 1: Table S1). To rule out assay-specific false positives, we counterscreened using a stably transfected reporter cell line containing a luciferase reporter for human SSPN promoter activity (hSSPN-luc). The top 1000 hits were rescreened in both hSSPN-EGFP (*n* = 3) and hSSPN-luciferase promoter reporter myotubes (*n* = 3). Of the 1000 hits, 63 compounds increased reporter expression in both reporter cell lines and were therefore considered confirmed hits. The confirmed hits were sorted into three groups based on common structural features: pharmacophore 1, pharmacophore 2, and the other category, which had no unifying structural features. Pharmacophore 2 compounds consisted of flat, multiring structures known to intercalate into DNA, which was considered a liability. We therefore focused on the pharmacophore 1 and other class of compounds.

### Hit-to-lead selection using dystrophin deficient murine and human muscle cells

For the initial hit-to-lead selection, all commercially available confirmed hits were tested at a concentration of 5.5 μM in dystrophin-deficient *mdx* myotubes to determine if the compounds were active in a relevant disease model. Within the pharmacophore 1 group, nine of the sixteen hits increased SSPN gene expression between 1.1- and 1.8-fold relative to the vehicle control (Fig. 1b). With the other group, eight of the twenty-three other hits increased SSPN gene expression between 1.2- and 2.0-fold (Fig. 1c). The initial hit-to-lead selection demonstrated that sequential screening with two separate reporter cell lines enables identification of compounds that increase SSPN mRNA levels in both wild-type and dystrophin-deficient murine muscle cells.

**Fig. 2:**
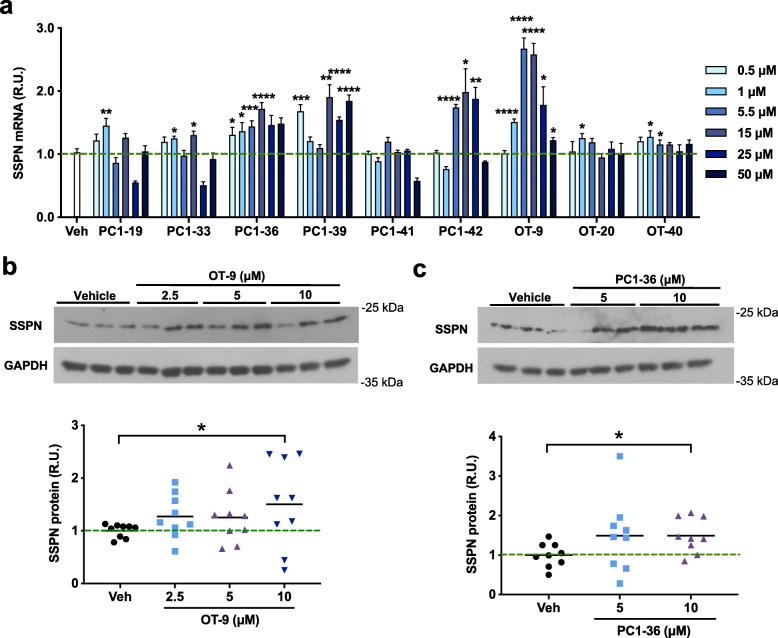
Confirmed hits increase sarcospan gene and protein expression in *mdx* myotubes. **a** Relative sarcospan gene expression in dystrophin-deficient H2K *mdx* cells treated with 0.5 to 50 μM of pharmacophore 1 or other compounds for 48 h. Gene expression was normalized to housekeeping gene β-Actin and vehicle-treated cells (0.2% DMSO). Data are represented as mean + SEM. *n* = 3–6. **b**–**c** Sarcospan immunoblot of dystrophin-deficient H2K *mdx* cells treated with 2.5 to 10 μM of compounds for 48 h. All cells were treated at day 2 of differentiation and harvested 48 h posttreatment. Sarcospan protein levels were quantified in ImageJ and normalized to GAPDH and vehicle-treated cells (0.2% DMSO). Cell lysates were probed between 2 and 4 times in independent western blots. Representative blots shown. Quantification shown below immunoblots includes all experiments. Data represents individual replicates and mean value. *n* = 3 per concentration. SSPN, sarcospan; R.U., relative units. *p < 0.05, **p < 0.01, ***p < 0.001, ****p < 0.0001

After excluding compounds that were unstable in solution or that produced highly variable results, nine compounds remained. These nine compounds were tested at six different concentrations ranging from 0.5 to 50 μM in *mdx* myotubes to determine the activity across a broad range of concentrations. All the compounds except PC1-41 demonstrated activity with at least one concentration (Fig. 2a). PC1-36, PC1-42, and OT-9 induced a concentration-dependent response that peaked at 5.5 μM. To determine if the increase in SSPN mRNA was also evident at the protein level, we treated *mdx* myotubes with 2.5 to 10 μM of OT-9, PC1-36, and PC1-42 and analyzed total protein lysates by immunoblotting with SSPN antibodies. We found that PC1-42 did not induce an increase in SSPN protein (data not shown). Compounds OT-9 and PC1-36 increased SSPN protein levels in the *mdx* myotubes by 1.5-fold, demonstrating that these compounds increased both SSPN gene and protein abundance in dystrophin-deficient muscle cells (Fig. 2b–c).

Previously, we profiled SSPN gene expression in differentiating myotubes and found that SSPN mRNA begins to increase on day 3 and reaches 10-fold increased levels on day 5 of differentiation, indicating that SSPN levels increase as cells differentiate [26]. To determine if the compound-induced increase in SSPN expression was due to enhanced differentiation, we treated *mdx* myotubes with OT-9 and assessed fusion index and expression of the myogenic transcription factor MYOG, markers of differentiation. OT-9 induced a slight increase in fusion index and MYOG gene expression in *mdx* myotubes, suggesting that the increase in SSPN may increase the rate of differentiation or that OT-9 may, in part, work through differentiation pathways (Additional file 2: Figure S1).

**Fig. 3:**
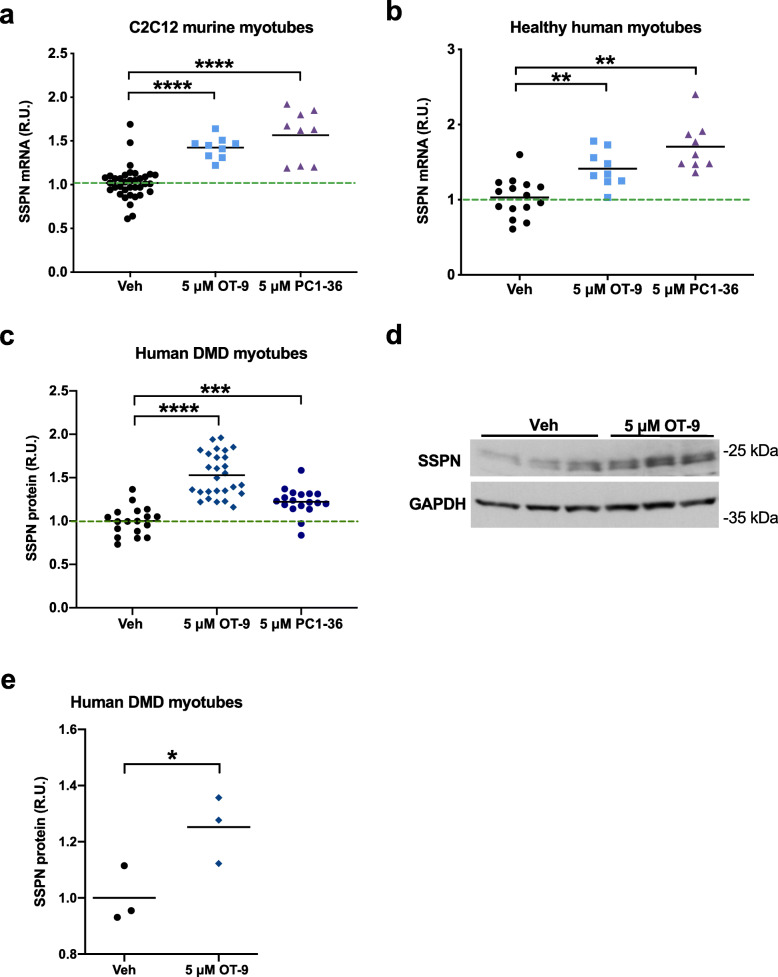
OT-9 increases sarcospan gene expression in mouse and human muscle cell lines and protein levels in human DMD myotubes. **a** C2C12 and **b** healthy human myotubes treated for 48 h with 5 μM of OT-9 and PC1-36 exhibit an increase in sarcospan gene expression. Gene expression was normalized to β-actin and vehicle-treated cells (0.1% DMSO). Human DMD myotubes treated with 5 μM of OT-9 or PC1-36 exhibit an increase in sarcospan protein levels as quantified by **c** an indirect sandwich ELISA and **d** immunoblot analysis. **e** Quantification of immunoblot results. Cells were treated on day 2 of differentiation and harvested after 48 h. R.U. = relative units normalized to protein concentration for the ELISA and GAPDH for the immunoblot analysis. Data represents individual replicates and mean value. *n* = 3–27. SSPN, sarcospan; R.U., relative units. *p < 0.05, **p < 0.01, ****p < 0.0001

To validate that the confirmed hits were not acting in a cell line–specific manner, we tested OT-9 and PC1-36 in wild-type C2C12 myotubes and immortalized myotubes from a healthy human patient. We found that the compounds increased SSPN mRNA levels in both cell lines, demonstrating that the hits worked across a range of cell lines (Fig. 3a–b). Treatment of C2C12 murine, healthy human, H2K WT murine, and H2K *mdx* murine myoblasts revealed that OT-9, but not PC1-36, increased SSPN mRNA levels in all myoblast lines. The unique ability of OT-9 to increase SSPN in myoblasts suggests that OT-9 and PC1-36 may have different biological targets (Additional file 3: Figure S2). To determine if increased myoblast proliferation accounted for the elevation in SSPN gene expression upon treatment, we quantified healthy human myoblasts after 24 h of treatment with 5 μM of OT-9 (Additional file 4: Figure S3). We found that OT-9 did not increase the number of cells per well and therefore concluded that the increase in SSPN gene expression was not due to increased proliferation.

To determine if the confirmed hits increase SSPN protein levels in human muscle cells, we first created an enzyme-linked immunosorbent assay (ELISA) capable of detecting changes in human SSPN levels across samples. Using recombinant human SSPN as a standard, we tested three pairs of commercially available antibodies that recognize different epitopes of the human SSPN polypeptide (Additional file 5: Figure S4a–b). The antibodies were either conjugated to the plate to capture SSPN protein (capture) or used to detect captured SSPN protein (detecting). The E2 capture + LS-N detecting (E2 cap + LS-N det) antibody combination produced the highest signal (Additional file 5: Figure S4c). We determined that using this antibody pair, the ELISA was capable of detecting as little as 1–2 pg of SSPN (Additional file 5: Figure S4d–e). Using the ELISA, we found that after 48 h of treatment with 5 μM of OT-9 and PC1-36 SSPN protein increased by 1.5- and 1.25-fold, respectively, in DMD myotubes (Fig. 3c). Confirmation by immunoblot analysis using SSPN antibodies revealed a 1.3-fold increase in SSPN protein abundance after 48 h of OT-9 treatment (Fig. 3d–e). We selected the more effective compound, OT-9, for further assessment using functional assays

### OT-9 compound increases laminin-binding adhesion complexes at the cell surface

**Fig. 4:**
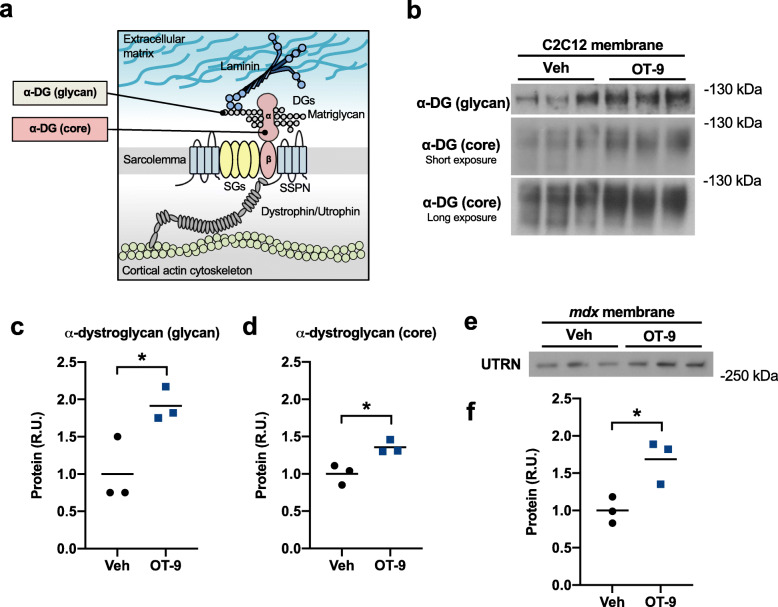
OT-9 increases laminin-binding adhesion complexes at cell surface. C2C12 myotubes treated with vehicle or 5 μM of OT-9 for 48 h were incubated in amine-reactive biotin to label cell surface proteins. Avidin was used to affinity purify the labelled proteins before immunoblot analysis with **a** antibodies recognizing the laminin-binding glycoepitope of alpha-dystroglycan (α-DG (glycan)) or core alpha-dystroglycan. **b** In cells treated with OT-9, both glycosylated alpha-dystroglycan and core alpha-dystroglycan were increased at the cell surface. **c**–**d** Quantification of immunoblots. **e** *mdx* myotubes treated with vehicle or 5 μM of OT-9 for 48 h were incubated in biotin to label cell surface proteins and affinity purified with avidin. Immunoblot analysis shows upregulation of utrophin associated with biotin-labelled cell surface proteins. **f** Quantification of utrophin immunoblot. Data represents individual replicates and mean value. *n* = 3. α-DG, alpha-dystroglycan; UTRN, utrophin; R.U., relative units. *p < 0.05

In striated muscle, SSPN is a scaffold for the three major laminin-binding adhesion complexes that connect the cell membrane (sarcolemma) to the extracellular matrix: the DGC, UGC, and α7β1D-integrin. Overexpression of SSPN in *mdx* muscle increases the localization of the UGC and α7β1D-integrin complex [22–24]. To determine if the OT-9-affects SSPN localization at the cell membrane in myotubes, we labeled cell surface proteins with an amine-reactive biotin. C2C12 myotubes treated with OT-9 were incubated in cell impermeable biotin, lysed to solubilize proteins, affinity purified with avidin, and eluted with LSB to obtain cell surface proteins. Using antibodies that recognize the laminin-binding glycoepitope of α-DG (glycan) and the core α-DG protein (Fig. 4a), we performed immunoblot analysis of the biotinylated cell surface proteins and total lysate. OT-9 increased glycosylated α-DG by 1.8-fold and core α-DG by 1.6-fold at the cell surface (Fig. 4b–d). Immunoblot analysis of total protein lysates revealed that OT-9 did not increase levels of glycosylated α-DG, but did increase core α-DG by 1.5-fold (Additional file 6: Figure S5). The difference in cell surface and total lysate levels of glycosylated α-DG suggests that OT-9 increased membrane localization of the laminin-binding α-DG.

In *mdx* mice overexpressing SSPN, the dystrophin paralogue utrophin is upregulated at the sarcolemma and contributes to the increase in membrane to ECM adhesion [22–24]. Using the same biotinylation experimental approach, we assessed whether OT-9 affected utrophin expression at the cell surface membrane. While utrophin is intracellular and therefore not directly biotinylated, the solubilization buffer contained a gentle detergent that preserved interactions within adhesion complexes, including that of utrophin, β-dystroglycan, and the cell surface α-DG. *mdx* myotubes treated with OT-9 exhibited a 1.6-fold increase in membrane-associated utrophin protein (Fig. 4e–f). Taken together, our findings demonstrate that OT-9 increased the sarcolemmal localization of both α-DG and utrophin, suggesting an upregulation of the laminin-binding utrophin glycoprotein complex.

### OT-9 improves membrane stability in dystrophin-deficient myotubes through upregulation of sarcospan

**Fig. 5:**
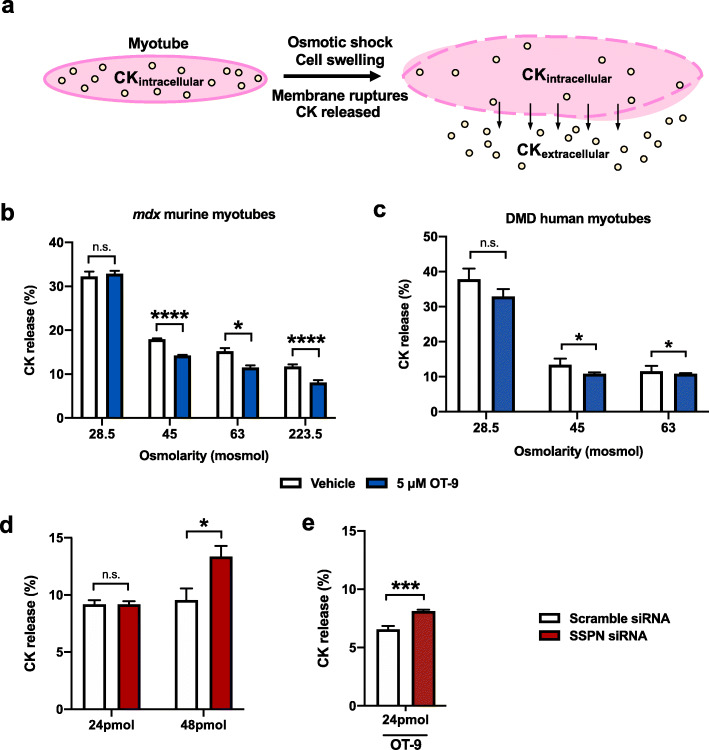
OT-9 improves membrane stability of dystrophin-deficient myotubes in part through upregulation of sarcospan. **a** The creatine kinase (CK) release assay entails subjecting myotubes to osmotic shock, which causes cell swelling and membrane damage, allowing for intracellular CK to be released from the cell into the surrounding medium. CK release is calculated by taking the ratio of CK_extracellular_/(CK_extracellular_ + CK_intracellular_). Day 2 **b** *mdx* and **c** DMD myotubes treated were treated for 48 h with 5 μM of OT-9 and subjected to osmotic shock with solutions ranging from 28.5 to 224.5 milliosmoles (mosmol). **d** *mdx* myotubes were transfected with 24 or 48 nM of scramble siRNA or siRNA targeting sarcospan. After 48 h, myotubes were subjected to osmotic shock with 45 mosmol solutions. The 24 nM SSPN siRNA transfection did not affect CK release relative to scramble control. The 48 nM concentration of sarcospan siRNA increased CK release relative to the scramble control, indicating sarcospan contributes to membrane stability regardless of treatment **e** *mdx* myotubes treated in parallel with 24 nM of sarcospan siRNA and 10 μM of OT-9 demonstrate that depletion of sarcospan reduced the ability of OT-9 to improve membrane stability. Data represents mean + SEM. *n* = 3. SSPN, sarcospan; R.U., relative units. *p < 0.05, **p < 0.01, ***p < 0.001, ****p < 0.0001

To determine if the increase in utrophin and dystroglycan at the cell membrane resulted in a functional improvement in membrane stability, we used a modified protocol for an in vitro creatine kinase (CK) release assay [32]. The assay entails subjecting myotubes to osmotic shock, which causes cell swelling and membrane damage, allowing for intracellular CK to be released from the cell into the surrounding medium (Fig. 5a). To determine the optimal conditions for osmotic shock that result in a detectable change in CK release, we treated *mdx* and DMD myotubes with vehicle or 5 μM of OT-9 for 48 h then induced osmotic shock using solutions ranging from 28.5 to 223.5 mosmol, with 223.5 mosmol being closest to physiological osmolarity (280 mosmol). Both *mdx* and DMD myotubes exhibited higher CK release with lower, more damaging osmolarity concentrations (Fig. 5b–c). Osmotic shock with the 28.5 mosmol solution resulted in a 30–40% CK release in both cell lines, while the 45 and 63 mosmol solution caused a 10–15% CK release, indicating relatively less membrane damage. In cells subjected to osmotic shock with 45, 63, and 223.5 mosmol solutions, OT-9 significantly reduced CK release, suggesting that OT-9 stabilized the membrane and protected it from osmotic shock-induced damage. Treatment with OT-9 did not reduce CK release in cells subjected to osmotic shock with 28.5 mosmol solutions, indicating that OT-9 was not able to stabilize the membrane likely due to severe membrane damage caused by the extremely low osmolarity.

To determine if the membrane stabilizing effect induced by OT-9 was dependent on SSPN, we performed siRNA-mediated knockdown of SSPN in parallel with compound treatment. To assess knockdown efficiency, we first treated *mdx* myotubes with 1, 5, or 10 μM of OT-9 and 24 nM of scramble siRNA or siRNA targeting SSPN mRNA (SSPN siRNA). In cells treated with vehicle control, SSPN siRNA reduced SSPN mRNA by 76% relative to the scramble control (Additional file 7: Figure S6). In cells treated with scramble siRNA and OT-9, SSPN mRNA increased by up to 1.4-fold relative to the vehicle control. In cells treated with SSPN siRNA and OT-9, SSPN levels were reduced relative to their respective scramble siRNA controls. However, even with SSPN siRNA, OT-9 increased SSPN mRNA levels due to the incomplete knockdown, leading us to use a higher siRNA concentration in subsequent experiments.

To assess the effect of SSPN knockdown on membrane stability in the absence of any compound treatment, we transfected *mdx* myotubes with 24 and 48 nM of scramble and SSPN siRNA and subjected the cells to osmotic shock with 45 mosmol solutions. In cells transfected with 24 nM of siRNA, knockdown of SSPN did not affect CK release (Fig. 5d). However, in cells transfected with 48 nM of siRNA, knockdown of SSPN increased CK release from 9 to 13%, indicating that loss of SSPN itself renders the sarcolemma more susceptible to membrane damage. To prevent confounding the data with changes in baseline CK release induced by SSPN knockdown, we selected the 24 nM siRNA concentration for following studies because it did not affect baseline CK release. We treated *mdx* myotubes with 10 μM of OT-9 in parallel with 24 nM of scramble or SSPN siRNA for 48 h prior to osmotic shock and discovered that depletion of SSPN increased the CK release from 6.5 to 8% (Fig. 5e). The results revealed that knockdown of SSPN reduced the ability of OT-9 to stabilize the sarcolemma, indicating that SSPN expression is required for the full membrane stabilizing effect of OT-9.

### Intramuscular injection of OT-9 increases SSPN gene expression in *mdx* mice

**Fig. 6:**
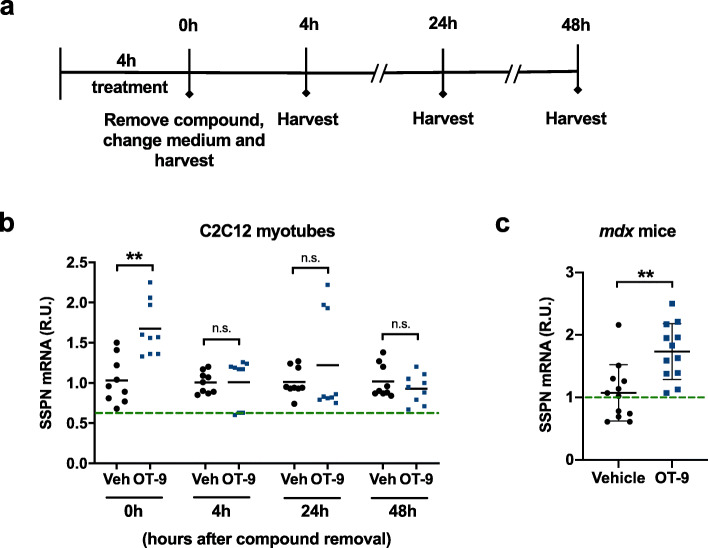
OT-9 increases sarcospan mRNA in vitro and in vivo in *mdx* muscle. **a** Schematic overview of experimental setup to test sarcospan mRNA stability in vitro. C2C12 myotubes were treated on the second day of differentiation with 5 μM of OT-9. After 4 h, the medium containing OT-9 was removed and changed to fresh medium without compound. The cells were harvested for gene expression analysis immediately after medium change (0 h), and 4 h, 24 h, and 48 h after. **b** OT-9 induced sarcospan gene expression after 4 h of treatment (0 h). After extended removal of the compound (4–48 h), sarcospan mRNA levels were the same in cells treated with vehicle and OT-9. **c** 20-week-old male *mdx* littermates were injected in both tibialis anterior muscles with vehicle (5% DMSO, 95% PBS) or 3 mg/kg  of OT-9. After 4 h, the muscles were harvested and processed for gene expression analysis. Gene expression was normalized to β-actin and vehicle-treated cells. Data represents individual replicates and mean value. *n* = 3 for in vitro and *n* = 2 for in vivo study. SSPN, sarcospan; R.U., relative units. **p < 0.01, ****p < 0.0001

After demonstrating the ability of OT-9 to increase SSPN protein and laminin-binding adhesion complexes at the membrane, which in turn led to improved membrane stability in dystrophin-deficient myotubes, we next interrogated the capacity of OT-9 to increase SSPN gene expression in vivo. Estimation of OT-9 stability revealed a large difference in half-life of the compound in mouse plasma (7.7 h) versus PBS (49 min) (Additional file 8: Table S2, Additional file 9: Table S3). Because cell culture medium is distinct from both solutions, we quantified SSPN mRNA stability in C2C12 myotubes treated with 5 μM of OT-9 for 4 h to determine if short-term treatment could induce SSPN expression. After 4 h of treatment with OT-9, medium containing the compound was removed and changed to fresh medium. The cells were harvested at 0, 4, 24, and 48 h after compound removal (Fig. 6a). Immediately after compound removal (0 h), SSPN mRNA levels were elevated by 1.5-fold over the vehicle control, demonstrating that OT-9 induces SSPN gene expression after just 4 h of treatment (Fig. 6b). However, 4, 24, and 48 h after compound removal, SSPN mRNA levels returned to baseline levels, which suggested that SSPN mRNA was upregulated for up to 4 h after induction by OT-9.

To evaluate the in vivo safety and activity of OT-9, we performed two pilot studies in wild-type C57/Bl6 and *mdx* mice. For the preliminary safety assessment of OT-9, we performed IP injections in 6-month-old C57/Bl6 males. The mice were injected with vehicle, 30 mg/kg of OT-9, or 50 mg/kg of OT-9 (*n* = 1). No signs of inflammation, necrosis, or compound accumulation were observed at the injection site. The liver, kidneys, pancreas, and intestine appeared to be of normal weight and size. As some insoluble particles were observed in 50 mg/kg solution of OT-9, the 30 mg/kg concentration was chosen for assessment of in vivo activity.

After the preliminary safety evaluation, we tested the ability of OT-9 to increase SSPN gene expression in vivo. We chose to assess activity after 4 h of treatment based on the finding presented in Fig. 6b, which demonstrated that 4 h of treatment with OT-9 was sufficient to significantly increase SSPN mRNA levels in vitro. Five 20-week-old male *mdx* littermates were subjected to intramuscular injections in both tibialis anterior (TA) muscles. Two mice were injected in both TAs with vehicle, and two mice were injected with 3 mg/kg of OT-9 (molar equivalent of 9.4 mM used in the safety assessment). After 4 h of treatment, the TAs were harvested and processed for gene expression analysis. No adverse effects were observed after local OT-9 injections in the mice. OT-9 induced a 1.7-fold increase in SSPN gene expression relative to the vehicle control–treated group, demonstrating that OT-9 is capable of increasing SSPN gene expression in *mdx* mice (Fig. 6c).

## Discussion

In summary, we identified a small molecule modulator of SSPN with in vitro and in vivo efficacy. OT-9 increased adhesion complex localization at the cell membrane, supporting our previously published results demonstrating that overexpression of SSPN in *mdx* mice increased the utrophin-glycoprotein complex at the sarcolemma [22, 24]. Intramuscular injections in *mdx* mice demonstrated that OT-9 increased SSPN gene expression in as little as 4 h, suggesting it quickly targets SSPN gene expression. However, a full pharmacokinetic profiling of OT-9 is part of the pipeline for developing OT-9 and determining its potential for in vivo efficacy. OT-9 induced SSPN protein by 1.5-fold levels. We previously investigated the levels of sarcospan necessary to rescue disease pathology in *mdx* mice through the availability of mouse lines expressing various levels of sarcospan [34]. We determined that mice overexpressing sarcospan by 1.5-fold were not rescued, while mice overexpressing sarcospan by 3-fold were rescued [34]. This demonstrated that the level of sarcospan overexpression needed to rescue *mdx* mice is somewhere between 1.5- and 3-fold.

Careful biochemical studies have revealed that the utrophin-glycoprotein complex is not a complete replacement of the dystrophin-glycoprotein complex due to differences in binding partners such as neuronal nitric oxide synthase and the actin cytoskeleton [35, 36]. However, upregulation of the utrophin-glycoprotein complex has been shown to be efficacious in numerous mouse studies (for review see [37]). Transgenic overexpression of utrophin to at least tenfold levels relative to *mdx* muscle resulted in a complete rescue of centrally nucleated fibers, peak force, and force drop in extensor digitorum longus, demonstrating it has strong therapeutic potential in vivo [38]. While upregulation of utrophin and sarcospan have overlapping pathways (i.e., activation during myogenic Akt signaling), there are features that distinguish sarcospan from utrophin. We have found that upregulation of sarcospan increases the α7β1D integrin complex, which has not been observed in mice overexpressing utrophin [34]. In addition to demonstrating that sarcospan overexpression improves skeletal and cardiac muscle function, we speculate that sarcospan overexpression has other beneficial effects and may act as a combinatorial therapy to boost existing therapies or therapies in development [24, 25, 39]. Recently, the group of Dr. Kay Davies found that transgenic utrophin overexpression, when used in combination with phosphonodiamidite morpholino antisense oligonucleotide to restore dystrophin, resulted in greater rescue than with exon skipping treatment alone [40]. Compared with exon skipping alone, *mdx* mice overexpressing utrophin and treated with antisense oligonucleotide exhibited greater resistance to force drop after eccentric contractions, an indicator of muscle integrity. The authors attributed the synergistic effect to the ability of utrophin to colocalize with dystrophin at the sarcolemma without competing for actin-binding partners.

As of 2020, there are three FDA-approved drugs for DMD and numerous therapies in the development phase or in clinical trials (for review see [41]). One of the main hurdles in drug development is translating results from animal to human studies. In 2018, the clinical trial for the small molecule utrophin modulator Ezutromid was terminated due to not meeting primary or secondary endpoints, which included upregulation of utrophin protein levels. After the termination of Ezutromid’s clinical trial, questions arose about the potential of utrophin upregulation as a target for DMD. However, follow-up studies point to poor physicochemical properties as the primary reason for Ezutromid’s lack of efficacy. Recent analysis of Ezutromid revealed that it was primarily cleared by cytochrome P450 1A (CYP1A) in the liver into less active metabolites [42]. In rats and pigs given multiple doses, Ezutromid increased biomarkers of CYP1A, further increasing its degradation. The development of Ezutromid highlights the many challenges of drug development that must be addressed during preclinical studies.

There has been a surge of major breakthroughs in mechanisms of disease and the emergence of new potential therapies including FDA approval for a gene therapy for spinal muscular atrophy and three FDA-approved drugs for DMD. While these have been tangible and exciting developments, there is much room for improvement of DMD treatments that significantly impact quality of life, lifespan, and have broad applicability. Exon-skipping drugs targeting dystrophin (i.e., Sarepta Therapeutics’s Eteplirsen targets exon 51, Golodirsen targets exon 53, Casimersen targets exon 45) are precision tools aimed at specific genetic mutations. Taken together, an estimated 60–80% of individuals with DMD possess mutations amenable to exon skipping, leaving 20–40% of patients in search of other treatments. In addition, recent clinical trials reveal serious immunological hurdles for AAV-based treatments. In 2020, a trial was halted in response to the death of two patients treated with an AAV-based therapy for myotubular myopathy (Audentes Therapeutics).

Several of the major benefits of SSPN as a target for DMD includes its potential to broadly treat patients, regardless of mutation, and the predicted lack of immune response due to SSPN being expressed endogenously in DMD patients. Another benefit of SSPN-based therapies is its potential to act as a combinatorial treatment to boost other modalities. Recently, the group of Dr. Kay Davies found that transgenic utrophin overexpression, when used in combination with phosphonodiamidite morpholino antisense oligonucleotide to restore dystrophin, resulted in greater rescue than with exon skipping treatment alone [40]. Compared with exon skipping alone, *mdx* mice overexpressing utrophin and treated with antisense oligonucleotide exhibited greater resistance to force drop after eccentric contractions, an indicator of muscle integrity. The authors attributed the synergistic effect to the ability of utrophin to colocalize with dystrophin at the sarcolemma without competing for actin binding partners.

Duchenne muscular dystrophy is 100% fatal and will always be present in the human population due to one-third of cases being caused by de novo mutations. We report the identification of a pharmacological upregulator of SSPN expression that possesses in vivo efficacy. Future studies will assess compound target, mechanism, and the potential for use as a combinatorial therapy to boost existing therapies and treatments in development. We believe that the multitude of efforts to identify treatments is necessary. In the recent years, private sector investment into therapeutics for DMD has increased significantly, opening the room for a variety of new modalities. Additionally, therapies initially developed for DMD may be repurposed for other diseases (examples: premature termination codon targeting therapies are used in cystic fibrosis, corticosteroids may be used for other myopathies).

## Conclusions

- High-throughput screening of over 200,000 compounds using cell-based promoter reporter assays for human SSPN gene expression identified three classes of pharmacophores that activate SSPN.
- The lead compounds increase SSPN gene and protein expression in dystrophin-deficient mouse and human myotubes.
- The lead compound OT-9 was further characterized and found to increase utrophin and dystroglycan at the cell surface.
- OT-9 increased membrane stability in dystrophin-deficient mouse and human myotubes, indicating its translatability across species.
- OT-9 increases SSPN gene expression in dystrophin deficient *mdx* mouse muscle, demonstrating its in vivo efficacy.

## Supplementary information

**Additional file 1: Table S1.** Plate quality. Robust strictly standardized mean difference (SSMD*) was used to assess plate quality and for hit selection.

**Additional file 2: Figure S1.** OT-9 increases differentiation in *mdx* myotubes. *mdx* myotubes were treated with 1, 5, and 10 μM of OT-9 on day 2 and assayed on day 4 of differentiation. (a-b) OT-9 induces slight increase in H2K *mdx* myotube differentiation as measured by fusion index and (c) Myogenin gene expression*.* Data represents individual replicates and mean value. n = 3. Scale bar = 200 μm. MYOG, myogenin; R.U., relative units. *p < 0.05, **p < 0.01.

**Additional file 3: Figure S2.** OT-9 is effective in multiple myoblast lines. C2C12, healthy human, H2K WT, and H2K *mdx* myoblasts are responsive to OT-9, but not PC1-36. Myoblasts were treated for 24 hours with 1, 5, and 10 μM of OT-9 or PC1-36. Gene expression was normalized to β-actin and vehicle-treated cells (0.1% DMSO). Data represents individual replicates and mean value. n = 3-6. SSPN, sarcospan; R.U., relative units. *p < 0.05, **p < 0.01, ***p < 0.001, ****p < 0.0001.

**Additional file 4: Figure S3.** OT-9 does not increase Mouly CTRL myoblast proliferation after 24 hours of treatment. Data represents individual replicates and mean value. n = 3.

**Additional file 5: Figure S4.** Development of an indirect sandwich ELISA to quantify human sarcospan protein. (a) Schematic of the topology of sarcospan in the sarcolemma. Sarcospan contains four transmembrane domains (TM1-4) and 2 extracellular loops (SEL: small extracellular loop; LEL: long extracellular loop or extracellular loop 2 (E-2)). (b) The commercially available antibodies against sarcospan used in the development of the ELISA target the N-term, E-2 loop, or full-length protein. (c) The standard curves using serially diluted recombinant human sarcospan (rhSSPN) protein demonstrates that the “E2 cap + LS-N det” antibody combination detects rhSSPN with the greatest sensitivity. (d) A standard curve generated using the E2 cap + LS-N det antibody combination and 0.002-0.1 ng/ml of rhSSPN. (e) A zoomed in version of the same standard curve illustrates that the ELISA can detect as little as 1-2 pg of rhSSPN.

**Additional file 6: Figure S5.** OT-9 increases laminin-binding adhesion proteins in total lysate. C2C12 myotubes treated with vehicle or 5 μM of OT-9 for 48 hours before immunoblot analysis. (a) Cells treated with OT-9 did not exhibit an increase in the fully glycosylated, laminin binding alpha-dystroglycan (α-DG (glycan)), but did exhibit an increase in core alpha-dystroglycan. GAPDH is shown as a loading control. (b-c) Quantification of immunoblots. Data represents individual replicates and mean value. n = 3. R.U., relative units normalized to GAPDH and vehicle control.

**Additional file 7: Figure S6.** siRNA-mediated knock down of SSPN results in a 76% knock down efficiency. *mdx* myotubes were treated in parallel with 1, 5, and 10 μM of OT-9 and 24 nM scramble control siRNA or siRNA targeting SSPN mRNA. Gene expression was normalized to β-actin and vehicle and scramble siRNA treated cells. Data represents mean + SEM. n = 3. SSPN, sarcospan; R.U., relative units. *p < 0.05, **p < 0.01, ****p < 0.0001.

**Additional file 8: Table S2.** Half-life of 1 μM of OT-9 and PC1-36 in CD-1 mouse plasma.

**Additional file 9: Table S3.** Half-life of 1 μM of OT-9 and PC1-36 in PBS pH 7.4.

## Data Availability

Not applicable.

## Abbreviations

α-DG: α-dystroglycan
BACT: β-actin
CK: creatine kinase
DMD: Duchenne muscular dystrophy/dystrophin
DGC: dystrophin-glycoprotein complex
DMSO: dimethyl sulfoxide
ECM: extracellular matrix
EGFP: enhanced green fluorescent protein
GAPDH: glyceraldehyde 3-phosphate dehydrogenase
hSSPN: human sarcospan
HTS: high-throughput screening
ITS: insulin transferrin selenium
MYOG: myogenin
SGCA: SSPN sarcospan
UTRN: utrophin
UGC: utrophin-glycoprotein complex
