# Supplementary figures and images for: High-throughput screening identifies modulators of sarcospan that stabilize muscle cells and exhibit activity in the mouse model of Duchenne muscular dystrophy
Source: Skelet Muscle. 2020 Sep 18;10:26. doi: 10.1186/s13395-020-00244-3 (PMC7499884; doi:10.1186/s13395-020-00244-3)

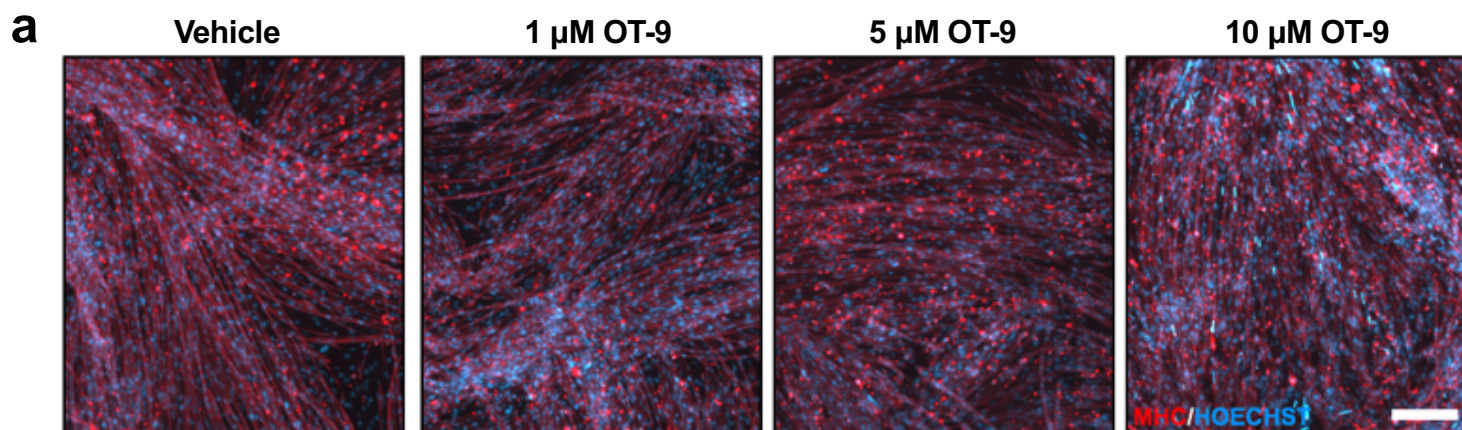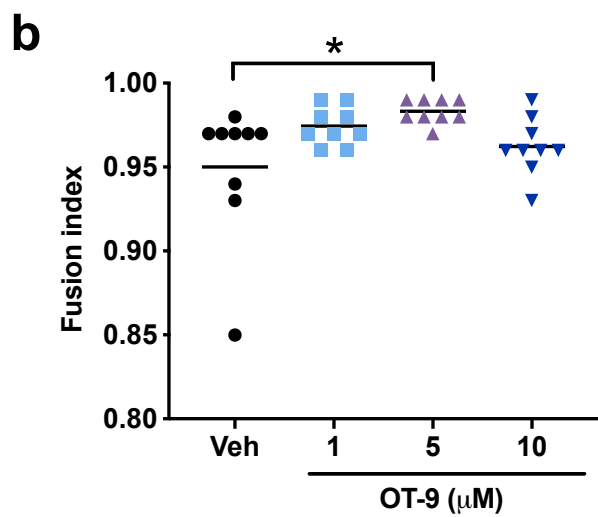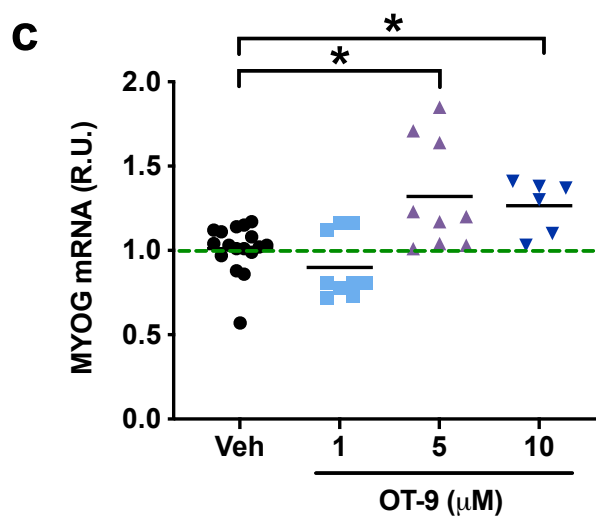

Supplement: Supplementary file 2 — Additional file 2: Figure S1. OT-9 increases differentiation in mdx myotubes. mdx myotubes were treated with 1, 5, and 10 μM of OT-9 on day 2 and assayed on day 4 of differentiation. (a-b) OT-9 induces slight increase in H2K mdx myotube differentiation as measured by fusion index and (c) Myogenin gene expression. Data represents individual replicates and mean value. n = 3. Scale bar = 200 μm. MYOG, myogenin; R.U., relative units. *p < 0.05, **p < 0.01. [file 13395_2020_244_MOESM2_ESM.pdf]

**a**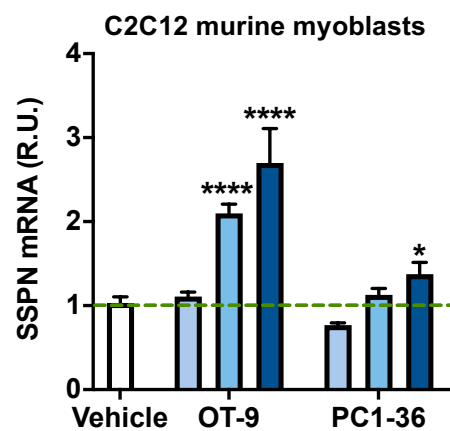**b**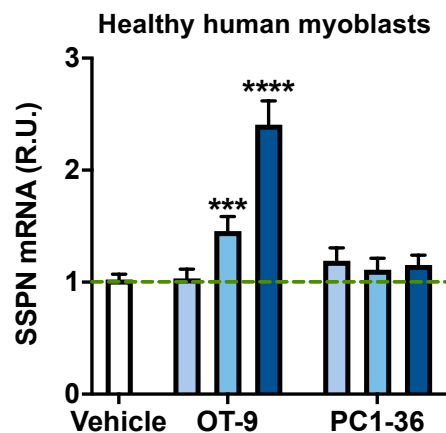**c**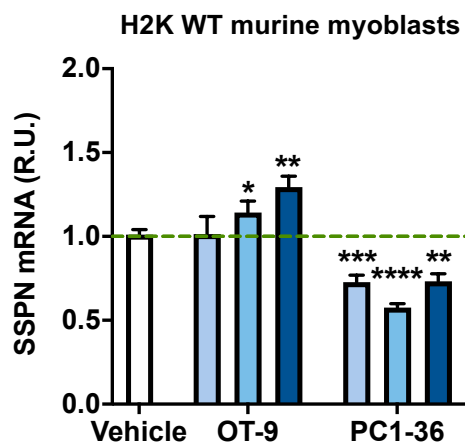**d**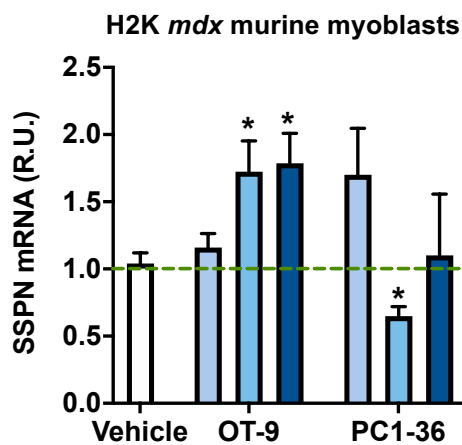

Supplement: Supplementary file 3 — Additional file 3: Figure S2. OT-9 is effective in multiple myoblast lines. C2C12, healthy human, H2K WT, and H2K mdx myoblasts are responsive to OT-9, but not PC1-36. Myoblasts were treated for 24 hours with 1, 5, and 10 μM of OT-9 or PC1-36. Gene expression was normalized to β-actin and vehicle-treated cells (0.1% DMSO). Data represents individual replicates and mean value. n = 3-6. SSPN, sarcospan; R.U., relative units. *p < 0.05, **p < 0.01, ***p < 0.001, ****p < 0.0001. [file 13395_2020_244_MOESM3_ESM.pdf]

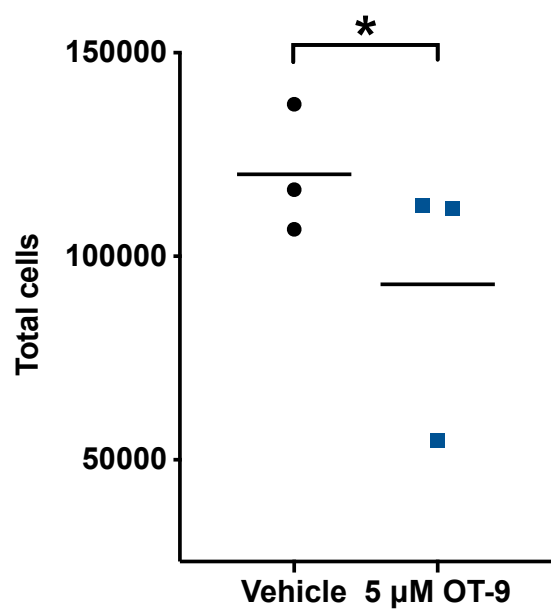

Supplement: Supplementary file 4 — Additional file 4: Figure S3. OT-9 does not increase Mouly CTRL myoblast proliferation after 24 hours of treatment. Data represents individual replicates and mean value. n = 3. [file 13395_2020_244_MOESM4_ESM.pdf]

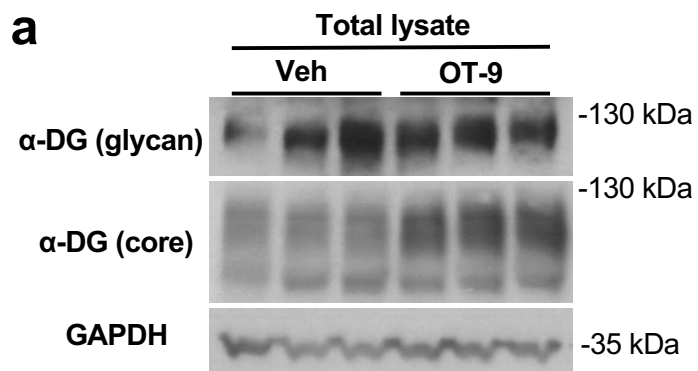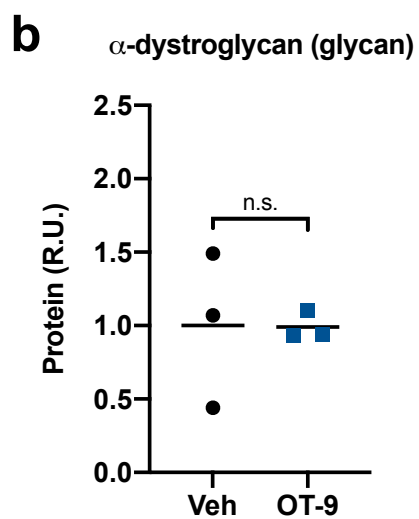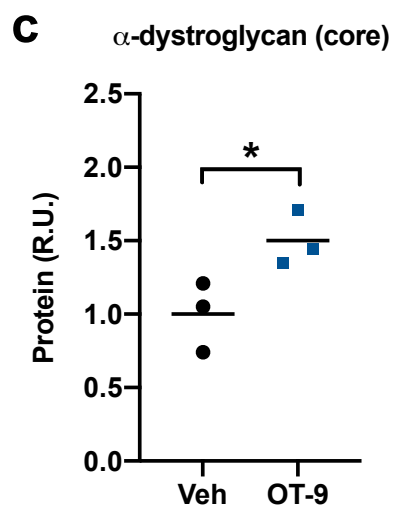

Supplement: Supplementary file 6 — Additional file 6: Figure S5. OT-9 increases laminin-binding adhesion proteins in total lysate. C2C12 myotubes treated with vehicle or 5 μM of OT-9 for 48 hours before immunoblot analysis. (a) Cells treated with OT-9 did not exhibit an increase in the fully glycosylated, laminin binding alpha-dystroglycan (α-DG (glycan)), but did exhibit an increase in core alpha-dystroglycan. GAPDH is shown as a loading control. (b-c) Quantification of immunoblots. Data represents individual replicates and mean value. n = 3. R.U., relative units normalized to GAPDH and vehicle control. [file 13395_2020_244_MOESM6_ESM.pdf]

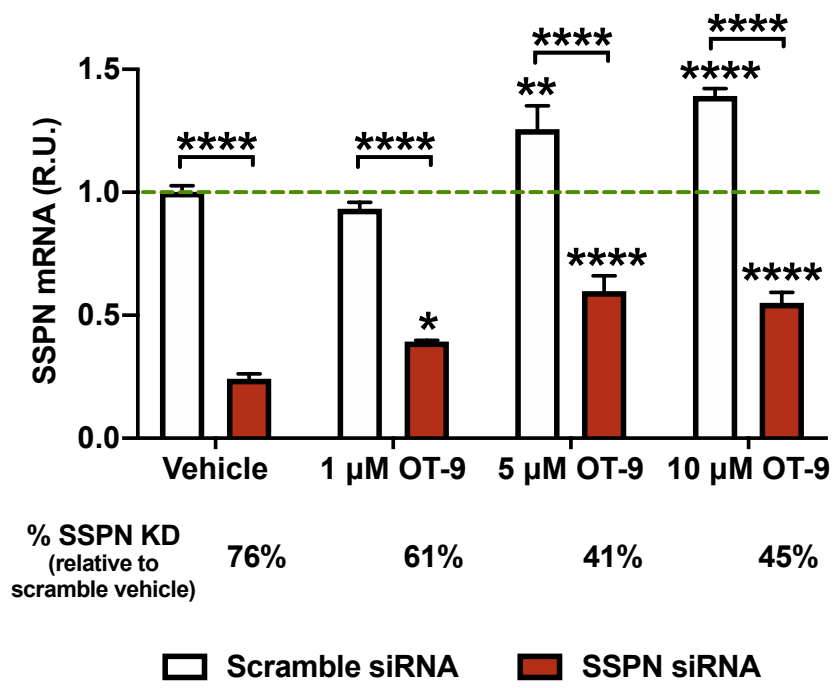

Supplement: Supplementary file 7 — Additional file 7: Figure S6. siRNA-mediated knock down of SSPN results in a 76% knock down efficiency. mdx myotubes were treated in parallel with 1, 5, and 10 μM of OT-9 and 24 nM scramble control siRNA or siRNA targeting SSPN mRNA. Gene expression was normalized to β-actin and vehicle and scramble siRNA treated cells. Data represents mean + SEM. n = 3. SSPN, sarcospan; R.U., relative units. *p < 0.05, **p < 0.01, ****p < 0.0001. [file 13395_2020_244_MOESM7_ESM.pdf]
