# Supplementary material for: High-throughput screening identifies modulators of sarcospan that stabilize muscle cells and exhibit activity in the mouse model of Duchenne muscular dystrophy
Source: Skelet Muscle. 2020 Sep 18;10:26. doi: 10.1186/s13395-020-00244-3 (PMC7499884; doi:10.1186/s13395-020-00244-3)

**a**

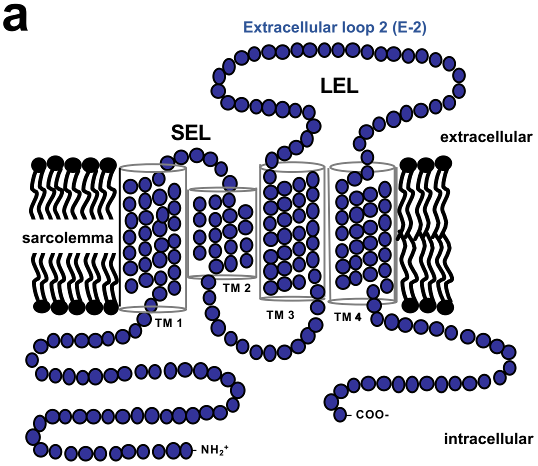


**b**

| Antibody | Company | Clonality | Host | Epitope |
|---|---|---|---|---|
| LS-N | LifeSpan BioSciences | polyclonal | rabbit | Human AA 1-60 (N-terminus) |
| E2 | Santa Cruz Biotechnology | monoclonal | mouse | Human AA 162-199 extracellular loop 2 (E-2) |
| NV | Novus Biologicals | polyclonal | mouse | Human AA 1-243 full length |

**c**

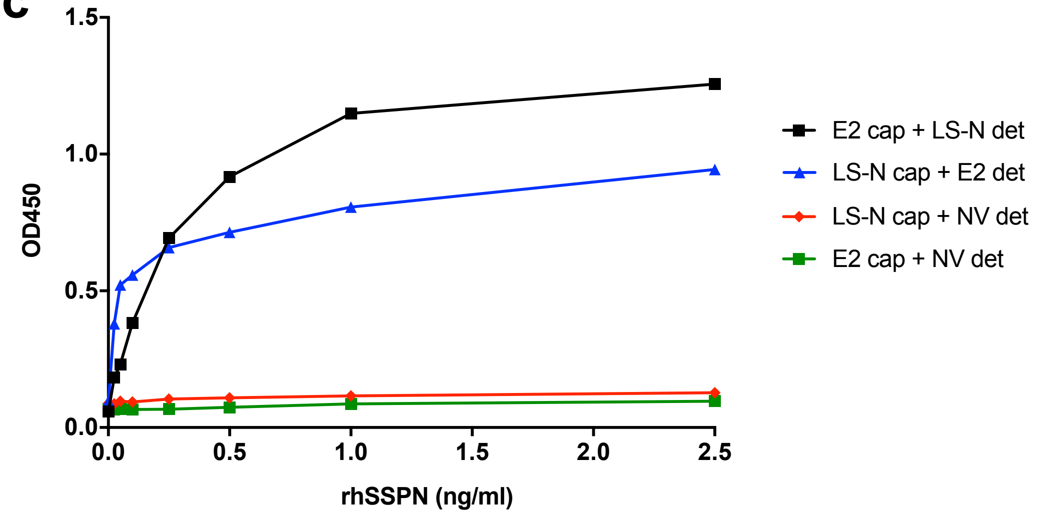


**d**

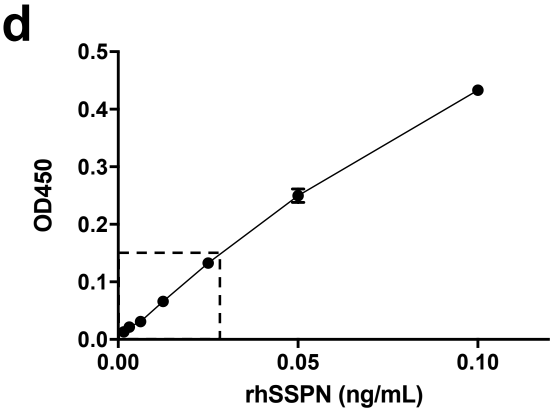


**e**

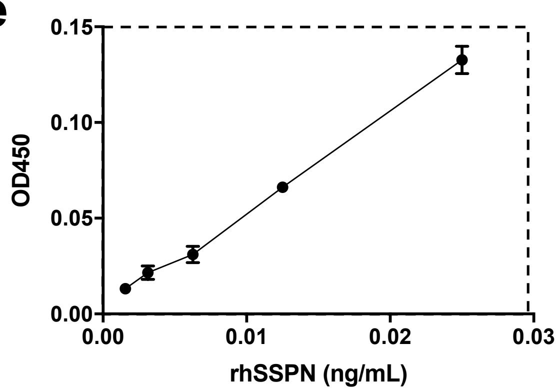

Supplement: Supplementary file 5 — Additional file 5: Figure S4. Development of an indirect sandwich ELISA to quantify human sarcospan protein. (a) Schematic of the topology of sarcospan in the sarcolemma. Sarcospan contains four transmembrane domains (TM1-4) and 2 extracellular loops (SEL: small extracellular loop; LEL: long extracellular loop or extracellular loop 2 (E-2)). (b) The commercially available antibodies against sarcospan used in the development of the ELISA target the N-term, E-2 loop, or full-length protein. (c) The standard curves using serially diluted recombinant human sarcospan (rhSSPN) protein demonstrates that the “E2 cap + LS-N det” antibody combination detects rhSSPN with the greatest sensitivity. (d) A standard curve generated using the E2 cap + LS-N det antibody combination and 0.002-0.1 ng/ml of rhSSPN. (e) A zoomed in version of the same standard curve illustrates that the ELISA can detect as little as 1-2 pg of rhSSPN. [file 13395_2020_244_MOESM5_ESM.pdf]
